# Public Understanding of Coercive Control in Northern Ireland

**DOI:** 10.1007/s10896-021-00355-5

**Published:** 2022-01-10

**Authors:** Susan Lagdon, Julie-Ann Jordan, Paula Devine, Mark A. Tully, Cherie Armour, Ciaran Shannon

**Affiliations:** 1 School of Psychology, Ulster University, Coleraine, Northern Ireland UK; 2 IMPACT Research Centre, Northern Health and Social Care Trust, Antrim, UK; 3 School of Social Sciences, Education and Social Work, Queen’s University, Belfast, UK; 4 School of Health Sciences, Ulster University, Coleraine, Northern Ireland UK; 5 School of Psychology, Queen’s University Belfast, Belfast, Northern Ireland UK

**Keywords:** Coercive Control, Psychological Abuse, Intimate Partner Violence, Domestic Abuse, Domestic Violent Crime

## Abstract

Coercive control is characterised by negative behaviours which intimidate, threaten, and humiliate a person or restrict a person’s liberty. In addition to being a known risk factor for experiencing other forms of violence, research has linked coercive control to symptoms of psychological distress and suicidality. In the UK, coercive and controlling behaviours within intimate and familial relationships have been legislated as offending behaviours. However, there still exists a lack of international evidence on wider public knowledge and understanding of coercive control. The Northern Ireland Life and Times Survey (NILT) is an annual cross-sectional representative survey of social policy topics. Participants are adults aged 18 years or over. Concerning coercive control, respondents were presented with two relationship scenarios: obvious and less obvious coercive control. Following each scenario, respondents indicated their level of agreement to ten statements covering attitudes towards coercive control, victims of coercive control, talking about coercive control, and whether coercive control is a crime. Respondents indicated whether they had heard of the term ‘coercive control’. Predictors of coercive control awareness were assessed using multinomial logistic regression. Mixed analysis of variance assessed if agreement levels to the ten statements varied by type of coercive control and victim gender. Most respondents said that they had heard of the term coercive control and knew what it meant. Those who had not heard of coercive control at all were more likely to be on a lower income, less qualified and younger, when compared to those who said they knew what the term meant. Significant interactions between coercive control type and victim gender were evident for all ten statements. While most respondents are aware of the term coercive control, a significant number have not and are therefore unlikely to recognise the signs of this type of abuse.

## Background

The concept of coercive control dates back many decades, perhaps most notably established by scholarly feminists who highlighted the significance of power and control within abusive relationships (Dobash & Dobash, 1979; Schechter, 1982; Smith, 1989). During this time, non-physical abuse including psychological and emotional exploitation of a victim, was recognised as one of the detrimental perpetrator tactics used to manipulate women into subordination. While coercive control may include acts of assault, it is not always physical in nature. Much of the early literature has explored this concept as behaviour among other forms of violence (Golding, 1999) and therefore the establishment of coercive and controlling behaviour in the absence of physical violence (and as a potentially independent abuse method), did not clearly appear until much later. What has been agreed is that coercive control is characterised by a pattern of negative behaviours which aim to intimidate, threaten and humiliate a person or restrict a person’s liberty (e.g. isolating a person from friends and family; taking control over aspects of everyday life such as where a person can go and who they can see; repeatedly putting a person down; making credible threats of violence; or economic oppression) entrapping them within an abusive cycle (Hamberger et al., 2017). The focus of the current work is coercive control in the absence of physical violence.

The evolution of coercive control as an abuse typology has coincided with wider understanding and theoretical debates on intimate partner violence (IPV) and abuse more broadly, including questions raised as to its gendered nature (Hamby, 2017; Straus, 1999, Afifi et al., 2009). Such controversy is also linked to limitations in how coercive control has been defined and measured (Hardesty et al., 2015) and in what context, particularly as it is underpinned by perpetrator motivation (Hamberger et al., 2017). While the evidence continues to demonstrate the elevated risk of partner violence and abuse among women, ever emerging research has demonstrated the experience of IPV victimisation among men also (Bates, 2020; Douglas & Hines, 2011; Tsui, 2014), although less is known about male’s experience of coercive control. Walby and Towers (2018) highlight distinctions in approaches to IPV to date which include gendered symmetric and asymmetric positions, ultimately offering a third option which is to focus on victim experience. Doing so is to also acknowledge the individual nature of the IPV experience (including patterns of coercive control) and therefore differences in the outcomes of such experiences (Nevala, 2017).

The negative consequence of IPV victimisation to both physical and mental health have been well established (Lagdon et al., 2014). The specific impact of coercive control has also been explored with research linking such abuse to symptoms of anxiety, depression and post-traumatic stress disorder (Anderson, 2008, Johnson & Leone, 2005, Lagdon et al., 2014) as well as suicidal behaviour (Coker et al., 2002). Coercive control is also associated with an increased risk of further violence exposure (Stark & Hester, 2019). For example, Dichter et al. (2018) explored the experience of female victims of IPV attending emergency departments in the United States (US). Those who reported experiencing coercive control were also more likely to report experiencing physical and sexual violence in the previous three months compared to the no coercive control group. Moreover, qualitative research with survivors often emphasises the negative and lasting impacts to mental health as a result of coercive control which continue long after the abusive relationship has ended (Lagdon et al., 2015). It is also important to acknowledge the significant negative impacts of coercive control on children who have witnessed such behaviour (Callaghan et al., 2018).

In recognition of the seriousness of coercive control, a number of United Kingdom (UK) regions and Ireland have developed and implemented legislation which makes an offence of coercive and controlling behaviours within intimate and familial relationships. This pattern of behaviour may include a combination of physical and sexual violence, as well as psychological and emotionally abusive behaviours and tactics which would be considered by a reasonable person to likely have a serious effect on a person (Barlow et al., 2020). Following the implementation of legislation, reports of its successful implementation and uptake have been mixed. For example, in a recent UK government review of controlling or coercive behaviour (CCB) (May 2021) the number of CCB offences had significantly increased from the previous two years (2016/17 *n* = 4, 246 Vs 2019/20 *n* = 24, 856) suggesting that the offence is being used across the Criminal Justice System (UK Home Office, 2021). That said, whilst recorded CCB offences had increased, those actually being charged with the offence had decreased with evidential difficulties cited as the main barrier to progression (*ibid*). Cases that had been prosecuted mainly involved other co-occurring offences such as violence against the person. The report authors provide research recommendations which included the need for better robust estimates and characterisation of coercive control as well as further awareness raising and understanding of coercive control among the general population. If those working within police and prosecution services are finding it difficult to recognise and evidence coercive control, so too will victims who are therefore unlikely to continue to report such experiences, particularly in the absence of physical harm (Crossman and Hardesty, 2018).

Adding further complexity is the potential gendered biases within help seeking and response to victims. Using the US National Intimate Partner and Sexual Violence Survey, Cho et al. (2020) investigated gender differences in survivor help-seeking, particularly with regards to formal (e.g. police and medical services) and informal (family and friends) sources. Perhaps unsurprising, the researchers found that females were more likely to seek formal help when victimised compared to males and that males tend to rely on informal sources. Further, those who had experienced multiple types of abuse were more likely to seek help than those who had experienced psychological violence only. Such findings reflect much of the discussion within the theoretical literature regarding the challenges of meeting the needs of victims of coercive control and the intersectional barriers faced by many when seeking formal support (Donovan & Barnes, 2020; Monterrosa, 2019; Parry & O'Neal, 2015). It is also important to mention the key role of informal support sources in not only aiding victims but also in mediating abuse experiences through early identification or acting as a lynchpin to specialist services if, and when needed (Parker, 2015). As Barlow et al. (2020) state, “Put simply, the law does not exist in a vacuum” (p. 161); we must therefore also consider the need for awareness and response readiness beyond statutory bodies.

Domestic violence and abuse are global topics of concern with multiple public campaign approaches adopted to address the issue at a societal level. Keller et al. (2010) discuss the ‘unintended effects of domestic violence campaigns’ that can result from gender led approaches, addressing the issue after the harm has already occurred or reinforcing existing social distributions of knowledge by educating those who are already aware. Before progressing with public education as potentially a primary prevention effort as well as a secondary support awareness approach, information on current social attitudes and understanding should be sought, particularly when legislation change is aimed at better reflecting social circumstance.

### Study Aims

Currently there is a lack of international evidence on wider public knowledge and understanding of coercive control which can support and guide best practice in this regard. To address the need for evidence-based knowledge to improve public awareness and victim responding to coercive control, a module of questions was included in the 2020 Northern Ireland Life and Times Survey with the aim of capturing baseline measurable data on public understanding of coercive control within intimate relationships. The study also explored the impact of victim gender, respondent gender, and the obviousness of behaviours on the public’s attitude towards coercive control behaviours.

## Methods

The Northern Ireland Life and Times Survey (NILT) is an annual cross-sectional survey of adults aged 18 years or over living across Northern Ireland. Founded in 1998, the survey uses a two-stage sampling methodology due to the absence of an appropriate individual-level sampling frame. Firstly, a systematic random sample of 15,000 addresses was selected from the Postcode Address File (PAF) list of private addresses. This is the most up-to-date listing of private addresses in Northern Ireland. Secondly, one person aged 18 years or over was randomly selected in each household using the ‘next birthday’ rule. Each letter sent to the selected addresses clearly stated that only the person with the next birthday was eligible to complete the survey online.

All previous years of NILT were undertaken using Computer Assisted Personal Interviews (CAPI) within a face-to-face interview, followed by a short self-completion questionnaire. However, the public health restrictions in response to the COVID-19 pandemic necessitated a move to a multi-modal approach in 2020. The survey was designed to be as inclusive as possible, and participants could choose to complete the survey in one of several ways: an online questionnaire, a phone interview or a video call. Where possible, and within government COVID-19 guidelines, face-to-face interviewers also called directly to selected households to encourage participation.

The 2020 Northern Ireland Life and Times Survey involved 1,292 interviews with adults aged 18 years or over. 95% of respondents completed the online questionnaire, whilst 5% of respondents participated via a phone or video call interview. A pilot was undertaken from 8–15 October 2020, with the fieldwork being undertaken between 11 November and 8 December 2020. All respondents were offered a £15 shopping voucher as a thank you for taking part. Advance letters were sent to 15,000 households, of which 411 refused to participate. However, the number of respondents was limited by the amount of funding available for the shopping vouchers, and so the survey was closed when 1,300 questionnaires had been completed. Eight surveys were unusable, meaning that 1,292 took part and a response rate of 9%. Full technical details are available at Devine (2021). The data were weighted to allow for disproportionate household size, reflecting the sampling methodology.

### Ethics

The 2020 Northern Ireland Life and Times Survey received ethical approval from the Ethics Committee of the School of Social Sciences, Education and Social Work, Queen's University Belfast, where the survey coordinator is based. Respondents were reminded that their participation was voluntary, and that they could withdraw at any time. The sensitive nature of the coercive control questions was also highlighted, and a link was provided to a leaflet providing appropriate sources of support.

### Measures

The survey includes questions on a range of social policy topics, with the range of topics changing each year in order to reflect contemporary social and policy debates. The questions on coercive control were informed by the findings of a consultation with a range of stakeholders with responsibility for relevant policy and service provision in NI. These were from both the statutory sector (such as Northern Health and Social Care Trust; Adult Mental Health and Children services; and Department of Justice NI), and non-governmental organisation sector (including Causeway Women’s Aid; Barnardo’s NI; and Nexus NI).

**Fig. 1:**
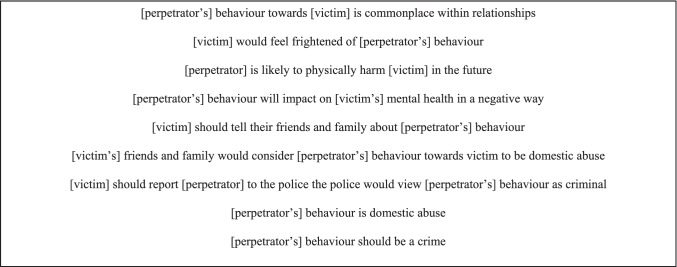
Attitudes towards coercive control—statement for response

NILT respondents were presented with two relationship scenarios focusing on a type of coercive control within intimate heterosexual relationships: obvious coercive control (scenario 1) and less obvious coercive control (scenario 2) (Full module details can be accessed from https://www.ark.ac.uk/nilt/2020/Coercive_Control/). Respondents were randomly assigned to one of two groups. Group A were presented with two scenarios involving a female victim and a male perpetrator. The two scenarios presented to Group B involved a male victim and a female perpetrator. Following each scenario, respondents indicated their level of agreement or disagreement using a 5-point scale (1 = strongly disagree, 2 = disagree, 3 = neither agree nor disagree, 4 = agree and 5 = strongly agree) to ten statements covering attitudes towards: coercive and controlling behaviours; victims of coercive control; talking about coercive control; and whether coercive control is a crime (See Fig. 1 for overview). Respondents were also asked if they had previously heard of the term ‘coercive control’, with response options ‘yes, and I know what it means’, ‘yes, but I am unsure what it means’ and ‘no’. For all questions in this module, respondents also had the option to say that they ‘don’t know’, or that they ‘prefer not to say’.

### Demographic Profile and Diversity

Of the 1,292 respondents, 770 were female (59% weighted) and 519 were male (41% weighted). Responses were not limited to binary options, and two respondents said that they would describe themselves ‘in another way’. However, the ‘in another way’ response was excluded from analysis due to the small number of respondents. Respondents’ age was recoded into a 6-way classification: 18–24-year (8%) 25–34 years (18%), 35–44 years (18%), 45–54 years (20%), 55–64 years (19%), and 65 years or more (17%). 28% of respondents were Catholic, 41% were Protestant, and 27% had no religion. In addition, 13 respondents (1%) were from non-Christian religions. 4% of respondents said that they considered themselves to be a member of a minority ethnic community. The small number of respondents within specific groups (e.g. non-Christian) reflect the wider religious profile of NI, but unfortunately means that the number of respondents is too small to include in statistical analysis. The majority of respondents (93%) reported that they were heterosexual, with 3% saying that they were gay or lesbian, and 2% saying that they were bisexual.

Respondents were asked to identify their highest educational qualification, and this was recoded into the following categories: degree level or higher (54%), higher education (11%), A level or equivalent (8%), GCSE grades A to C or equivalent (16%), GCSE grades D to G or equivalent (7%), other qualifications (3%), and no qualifications (4%). For the purposes of the multivariate analysis, this variable was reverse coded ranging from 1 (no qualifications) to 6 (degree level or higher), and the ‘other qualification’ and ‘don’t know’ responses were excluded. Self-reported income level responses were 1: low income (7%), 2: middle income (62%) and 3: high income (28%), with 4% indicating that they didn’t know. An urban/rural indicator, as defined by the Northern Ireland Statistics and Research Agency, was applied based on the respondent’s postcode.

Legal relationship status was assigned based on responses to a question on legal status: 1: single (never married) (28%); 2: married and living with spouse (59%); 3: civil partner in legally-registered partnership (1%); 4: married and separated from spouse (3%); 5: divorced (6%); 6: widowed 4%): 7: in legally-recognised civil partnership and separated from civil partner (0%); 8: formerly a civil partner, the civil partnership now legally dissolved (0%); 9: surviving civil partner as partner having since died (9%). Those respondents giving responses 2 or 3 (59%) were classified as in a legal relationship.

The results of the most recent Census of Population (undertaken in April 2021) are not yet available, and so comparison must be made with appropriate robust large-scale sample surveys. The demographic profile of the NILT respondents reflects the profile of the wider population as evidenced by the Continuous Household Survey (CHS) survey (https://www.nisra.gov.uk/publications/chs-results).

### Data Analysis

All analyses were conducted in SPSS Statistics Version 26 and were weighted by household size to reflect the sampling methodology. The demographic profiles of Samples A (female victim) and B (male victim) were compared using chi-squared tests for gender, income, urban/rural and legal relationship, and via independent samples t-tests for the age and qualification variables.

Multinomial logistic regression models were used to examine predictors of coercive control awareness. The reference category was ‘yes, and I know what it means’, and this was compared against ‘yes, but I am unsure what it means’, and ‘no’, responses. ‘Don’t know’ responses to the awareness of coercive control question were excluded from the multinomial regression models as their rarity (1%) increased the risk of biased estimates. Predictors included in the models were age, income, qualifications, legal relationship (reference = in a legal relationship), gender (reference = female), and urban/rural (reference = urban). Income and qualification variables were treated as continuous by excluding the small proportion of people who ticked ‘don’t know’ to the income question (4%) and those who ticked ‘other’ to the qualification question (3%). There were < 0.1% ‘don’t know’ responses to the age, gender, legal relationship and urban/rural questions; such responses were excluded from the analyses.

Mixed analysis of variance models assessed if agreement levels to the ten statements varied by type of coercive control (obvious vs less obvious) and victim gender (female vs male) and respondent gender (female vs male). In these models ‘don’t know’ responses were excluded so that the dependent variables could be treated as continuous. Separate analyses were conducted to examine if victim gender was associated with the likelihood of responding ‘don’t know’ to the statements presented in the obvious and less obvious coercive control scenarios.

Listwise deletion was used in all analyses as levels of missing data on the coercive control questions (0.4–0.7%) and demographic questions (0.0–2.1%) were very low.

## Results

### Sample Demographics

Descriptive statistics for Samples A and B are displayed in Table 1. Chi-squared tests and independent samples t-tests revealed no significant differences between the demographic profiles of Samples A and B.

**Table 1:** Demographics for both Samples A and B

		Sample A (female victim)		Sample B (male victim)		Sample A vs Sample B	
		mean/n	sd/%	mean/n	sd/%	t/x^2^	p
Age	46.87	16.16	48.37	15.97	-1.67	.095	
Qualification	4.74	1.60	4.82	1.53	.84	.403	
Gender identity	Male	266	40.49%	257	40.61%	< .01	.967
	Female	391	59.51%	376	59.39%		
Income	Low income	197	31.55%	154	25.96%	4.78	.091
	Middle income	382	61.31%	396	66.87%		
	High income	45	7.14%	42	7.17%		
Urban/rural	Rural	236	35.82%	225	35.51%	.02	.887
	Urban	422	64.18%	409	64.49%		
Legal Relationship	Yes	385	58.71%	378	60.32%	.34	.560
	No	271	41.29%	249	39.68%		

### Awareness of Coercive Control

The majority of respondents said that they had heard of the term coercive control and knew what it meant (*n* = 811; 62.9%). The remainder had heard of coercive control but did not know what it meant (*n* = 260; 20.1%); had never heard of it (*n* = 205; 15.9%) or responded ‘don’t know’ (*n* = 14; 1.1%). Predictors of coercive control awareness were examined via multinomial logistic regression (Table 2). Being younger and male were both associated with a greater likelihood of responding ‘yes, but I am unsure what it means’ as opposed to ‘yes, and I know what it means’. Those who had not heard of coercive control at all were more likely to be on a lower income, less qualified and younger, when compared to those who said they knew what the term meant.

**Table 2:** Multinomial logistic regression model predicting awareness of coercive control

Coercive Control Awareness category	Predictor	b	SE	Wald (df)	p	OR
Yes, but I am unsure what it means	Income	-0.24	0.15	2.63 (1)	.105	0.79
	Age	-0.02	0.01	9.05 (1)	.003	0.98
	Qualification	-0.06	0.05	1.06 (1)	.302	0.95
	Gender (male)	0.34	0.16	4.55 (1)	.033	1.40
	Urban/rural (rural)	-0.01	0.16	0.01 (1)	.931	0.99
	Legal relationship (no)	0.05	0.17	0.08 (1)	.784	1.05
No	Income	-0.42	0.17	5.92 (1)	.015	0.66
	Age	-0.05	0.01	45.05 (1)	.000	0.96
	Qualification	-0.29	0.06	24.99 (1)	.000	0.75
	Gender (male)	0.17	0.19	0.85 (1)	.356	1.19
	Urban/rural (rural)	0.16	0.18	0.78 (1)	.376	1.18
	Legal relationship (no)	0.02	0.20	0.01 (1)	.936	1.02

*Cox & Snell = .09; Nagelkerke = .11; McFadden = .05*

### Attitudes Towards Obvious and Less Obvious Coercive Control

Mean agreement scores to the coercive control statements by coercive control type and victim gender are shown in Table 3. Mixed analysis of variance models (victim gender by coercive control type) for each of the ten statements are presented in Table 4. Significant interactions between coercive control type and victim gender were evident for all ten statements. Independent samples t-tests were used to further explore the significant interactions. When the abuse described was obvious, agreement to seven of the ten statements was significantly higher, if the victim was portrayed as female rather than male: *frightened* (t (1267) = 4.54, p < 0.001, d = 0.27); *physical harm* (t (1250) = 3.33, p = 0.001, d = 0.19); *tell friends & family* (t (1264) = 3.37, p = 0.001, d = 0.19); *friends/family consider it domestic abuse* (t (1257) = 3.29, p = 0.001, d = 0.17); *report to police* (t (1255) = 4.07, p < 0.001, d = 0.22); *police view behaviour as criminal* (t (1194) = 3.34, p = 0.001, d = 0.19); *behaviour should be a crime* (t (1258) = 2.37, p = 0.018, d = 0.13). All of these effects were small in nature (Cohen, 1988). Agreement levels did not differ significantly between the female and male victim conditions in the obvious scenarios for the statements relating to the behaviour being *commonplace*, affecting *mental health,* and *being domestic abuse*.

**Table 3:** Mean agreement levels to the survey statements by coercive control type and victim gender

Survey statements	Obvious coercive control				Less obvious coercive control				Obvious coercive control				Less obvious coercive control			
	Female victim		Male victim		Female victim		Male Victim		Female Respondent		Male Respondent		Female Respondent		Male Respondent	
	Mean	SD	Mean	SD	Mean	SD	Mean	SD	Mean	SD	Mean	SD	Mean	SD	Mean	SD
1. Commonplace	2.21	1.08	2.28	1.10	2.46	1.10	2.77	1.06	2.28	1.14	2.20	0.99	2.60	1.12	2.64	1.04
2. Frightened	4.83	0.44	4.70	0.54	4.11	0.91	3.46	1.01	4.83	0.43	4.68	0.56	3.92	0.97	3.59	1.04
3. Physical harm	4.71	0.56	4.60	0.65	3.71	1.08	3.03	1.03	4.72	0.57	4.57	0.65	3.51	1.08	3.16	1.12
4. Mental health	4.85	0.40	4.84	0.41	4.64	0.62	4.35	0.72	4.89	0.34	4.78	0.48	4.64	0.58	4.29	0.78
5. Tell friends & family	4.80	0.45	4.70	0.60	4.58	0.63	4.19	0.81	4.81	0.46	4.66	0.61	4.52	0.68	4.19	0.81
6. Friends/family consider it domestic abuse	4.80	0.44	4.71	0.60	4.26	0.86	3.67	1.07	4.80	0.50	4.69	0.56	4.08	1.01	3.81	1.00
7. Report to police	4.73	0.55	4.59	0.70	3.46	1.15	2.83	1.05	4.75	0.53	4.54	0.74	3.30	1.14	2.92	1.11
8. Police view behaviour as criminal	4.37	0.91	4.19	0.97	2.98	1.22	2.50	1.02	4.39	0.89	4.13	0.99	2.85	1.19	2.56	1.07
9. Behaviour is domestic abuse	4.85	0.42	4.83	0.45	4.34	0.86	3.84	1.02	4.88	0.38	4.78	0.49	4.25	0.89	3.86	1.05
10. Behaviour should be a crime	4.75	0.57	4.67	0.62	3.79	1.07	3.14	1.11	4.79	0.53	4.59	0.67	3.68	1.11	3.16	1.11

**Table 4:** Mixed Analysis of Variance models for the survey statements: Victim gender by coercive control type

Survey statements	Victim Gender			Coercive Control type			Victim gender * Coercive Control type		
	f (df)	p	ɳp^2^	F (df)	p	ɳp2	F (df)	p	ɳp2
1. Commonplace	11.53 (1,1314)	.001	.01	195.86 (1,1314)	< .001	.13	27.88 (1,1314)	< .001	.02
2. Frightened	172.49 (1,1471)	< .001	.11	1476.56 (1,1471)	< .001	.50	113.95 (1,1471)	< .001	.07
3. Physical harm	148.51 (1,1380)	< .001	.10	1891.63 (1,1380)	< .001	.58	98.09 (1,1380)	< .001	.07
4. Mental health	46.39 (1,1501)	< .001	.03	455.68 (1,1501)	< .001	.23	78.94 (1,1501)	< .001	.05
5. Tell friends & family	84.87 (1,1493)	< .001	.05	418.01 (1,1493)	< .001	.22	78.25 (1,1493)	< .001	.05
6. Friends/family consider it domestic abuse	134.12 (1,1456)	< .001	.08	1012.74 (1,1456)	< .001	.41	109.73 (1,1456)	< .001	.07
7. Report to police	117.75 (1,1420)	< .001	.08	2403.71 (1,1420)	< .001	.63	71.37 (1,1420)	< .001	.05
8. Police view behaviour as criminal	51.45 (1,1342)	< .001	.04	2091.76 (1,1342)	< .001	.61	26.92 (1,1342)	< .001	.02
9. Behaviour is domestic abuse	87.45 (1,1470)	< .001	.06	980.03 (1,1470)	< .001	.40	99.43 (1,1470)	< .001	.06
10. Behaviour should be a crime	106.11 (1,1434)	< .001	.07	1885.10 (1, 1434)	< .001	.57	101.81 (1,1434)	< .001	.07

For the less obvious abuse scenarios, agreement was higher when the victim was described as female as opposed to male for nine of the ten statements: *frightened* (t (1245) = 12.02, p < 0.001, d = 0.68); *physical harm* (t (1175) = 11.13, p < 0.001, d = 0.64); *mental health* (t (1266) = 7.60, p < 0.001, d = 0.43); *tell friends & family* (t (1262) = 9.43, p < 0.001, d = 0.54); *friends/family consider it domestic abuse* (t (1233) = 10.68, p < 0.001, d = 0.61); *report to police* (t (1205) = 9.92, p < 0.001, d = 0.57); *police view behaviour as criminal* (t (1174) = 7.42, p < 0.001, d = 0.43); *behaviour is domestic abuse* (t (1239) = 9.20, p < 0.001, d = 0.53); *behaviour should be a crime* (t (1219) = 10.46, p < 0.001, d = 0.60). Generally speaking, these effect sizes correspond most closely with a medium sized effect (Cohen, 1988). In the less obvious scenarios for one statement, *commonplace*, agreement levels were actually higher in the male victim condition than the female victim condition (t (1149) = 4.75, p < 0.001, d = 0.29).

‘Don’t know’ responses constituted < 5% of responses to the statements presented in the obvious coercive control scenario, with the exception on *commonplace* (10.8%) and police view behaviour as criminal (6.9%). In the less obvious coercive control ‘don’t know’ represented < 5% of responses for five of the ten statements presented, and were somewhat higher for *commonplace* (10.3%), *physical harm* (8.3%), *report to police* (6%), *police view behaviour as criminal* (8.5%), and *behaviour should be a crime* (5.1%). Chi-squared difference tests showed no significant association between victim gender and the tendency to respond ‘don’t know’ in the obvious coercive control scenarios. In less obvious coercive control scenarios, for three of the ten statements a greater proportion of respondents chose ‘don’t know’ when the victim was female as opposed to male: *physical harm* (9.9% vs 6.8%; x^2^ (1) = 4.00, p = 0.045); *report to police* (8.1% vs 3.9%; x^2^ (1) = 9.66, p = 0.002); *police view behaviour as criminal* (11.6% vs 5.4%; x^2^ (1) = 15.80, p < 0.001).

Mean agreement scores to the coercive control statements by coercive control type and respondent gender are displayed in Table 3. Mixed analysis of variance models (respondent gender by coercive control type) for each of the ten statements are presented in Table 5. Only the main effects were significant for *police view behaviour as criminal,* reflecting generally higher levels of agreement for the obvious scenarios and for female respondents. The interaction between respondent gender and coercive control type was significant for the other statements. While the interaction effect for *commonplace* was significant, the independent samples t-tests used to explore this interaction showed no significant difference between males and females for the obvious and the less obvious conditions. A significant interaction was also evident for *report to police;* however, the effect size was negligible*.* For the other seven statements, in both the obvious and less obvious coercive control scenarios, female respondents expressed higher levels of agreement than males. The gender difference was greater for the less obvious than the obvious scenarios as evidenced by visual inspection of the interaction plots and the independent t-tests used to unpick the interactions: *frightened*—*obvious* (t (1265) = -5.21, p < 0.001, d = 0.31); *frightened* – less *obvious* (t (1243) = -5.68, p < 0.001, d = 0.33); *physical harm*—*obvious* (t (1248) = -4.19, p < 0.001, d = 0.25); *physical harm* – less *obvious* (t (1173) = -5.38, p < 0.001, d = 0.32); *mental health*—*obvious* (t (1269) = -4.86, p < 0.001, d = 0.27); *mental health* – less *obvious* (t (1264) = -9.31, p < 0.001, d = 0.53); *tell friends & family*—*obvious* (t (1262) = -4.75, p < 0.001, d = 0.29); *tell friends & family* – less *obvious* (t (1260) = -7.81, p < 0.001, d = 0.45); *friends/family consider it domestic abuse*—*obvious* (t (1255) = -3.44, p = 0.001, d = 0.21); *friends/family consider it domestic abuse* – less *obvious* (t (1231) = -4.62, p < 0.001, d = 0.27); *behaviour is domestic abuse*—*obvious* (t (1266) = -4.19, p < 0.001, d = 0.23);

**Table 5:** Mixed Analysis of Variance models for the survey statements: Respondent gender by coercive control type

Survey statements	Respondent Gender			Coercive Control type			Respondent gender * Coercive Control type		
	f (df)	p	ɳp^2^	F (df)	p	ɳp2	F (df)	p	ɳp2
1. Commonplace	0.39 (1,1311)	.533	.00	196.67 (1,1311)	< .001	.13	6.97 (1,1311)	.008	.01
2. Frightened	56.19 (1,1468)	< .001	.04	1357.42 (1,1468)	< .001	.48	11.68 (1,1468)	.001	.01
3. Physical harm	40.94 (1,1377)	< .001	.03	1746.21 (1,1377)	< .001	.56	11.10 (1,1377)	.001	.01
4. Mental health	104.14 (1,1498)	< .001	.07	483.07 (1,1498)	< .001	.24	51.61 (1,1498)	< .001	.03
5. Tell friends & family	70.82 (1,1490)	< .001	.05	419.28 (1,1490)	< .001	.22	24.83 (1,1490)	< .001	.02
6. Friends/family consider it domestic abuse	29.42 (1,1453)	< .001	.02	939.72 (1,1453)	< .001	.39	11.76 (1,1453)	.001	.01
7. Report to police	62.80 (1,1417)	< .001	.04	2250.28 (1,1417)	< .001	.61	6.02 (1,1417)	.014	.00
8. Police view behaviour as criminal	33.85 (1,1339)	< .001	.03	1966.27 (1,1339)	< .001	.60	0.26 (1,1339)	.609	.00
9. Behaviour is domestic abuse	63.65 (1,1467)	< .001	.04	958.36 (1,1467)	< .001	.40	34.27 (1,1467)	< .001	.02
10. Behaviour should be a crime	90.35 (1,1431)	< .001	.06	1791.28 (1,1431)	< .001	.56	23.80 (1,1431)	< .001	.02

*behaviour is domestic abuse* – less *obvious* (t (1237) = -7.15, p < 0.001, d = 0.41); *behaviour should be a crime* – *obvious* (t (1256) = -5.98, p < 0.001, d = 0.34); *behaviour should be a crime* – less *obvious* (t (1217) = -7.88, p < 0.001, d = 0.47).

## Discussion

Whilst there is a growing body of work geared towards conceptualisation and measurement of coercive control (Robinson & Myhill, 2021; Stark & Hester, 2019; Crossman & Hardesty, 2018; Donovan and Barnes, 2021; Walby & Towers, 2018), less empirical evidence is available with regards to wider public knowledge and attitude in relation to this. The experience of IPV will not typically start on day one of a relationship, with many victim accounts describing the development of an abusive pattern over time which can take months or years to be fully apparent (Lagdon et al., 2015, Williamson, 2010). Stark (2013) describes coercive control as the abuse ‘hidden in plain sight’, and its subtle emergence is its most dangerous advantage. If we are truly honest with ourselves, many of us, even the most informed, would find it difficult to pinpoint the beginning of the pattern.

The current research used scenarios of obvious and less obvious coercive control as a vehicle to engage with the public of NI regarding their knowledge and attitudes on coercive and controlling behaviours; victims of coercive control; talking about coercive control; and whether coercive control is a crime, ahead of legislation changes which will make an offence of this type of abuse. This methodological approach allowed for depersonalisation of a sensitive issue, as well as topic focus and opportunity for modification while maintaining consistency (Schoenberg and Ravdal, 2000), which in the current study relates to changes in gender of perpetrator and victim within the scenarios and presenting these to a split sample. As far as the authors are aware, the present study is the first to examine baseline levels of knowledge and attitudes towards coercive control with a focused geographical region prior to the introduction of legislation criminalising coercive control.

Our findings demonstrated that a significant proportion of the study sample had not heard of the term coercive control or did not know what it means (36%). Whilst it is encouraging that 63% had heard of the term and understood what it means, the present study did find that awareness rates were significantly lower than this for certain demographic groups; specifically, young people, males, those who are less qualified, and those from a low income background. These findings are perhaps unsurprising given the body of evidence which discusses the links between low income households, education and younger age as a vulnerability factor for IPV victimisation (Capaldi et al., 2012).

Moreover, findings also demonstrated greater levels of agreement across 7/10 statements for obvious and 9/10 for less obvious cases of coercive control when the victim was female rather than male. Taken together with the victim- and respondent-gender analysis, results suggest that the public are less knowledgeable about the experiences, impacts and support available to male victims in relation to coercive control, as well as difficulties in identifying early signs of abuse towards men. Within cases of obvious control, there tended to be strong agreement amongst the population sample that the described behaviours would leave a person feeling frightened, that their mental health would be impacted, that the victim is likely at risk of future physical harm, and should speak with friends and family. There was also a high level of agreement that the behaviour would be considered abusive, should be reported to the police and should be a crime. The level of agreement to all statements presented notably reduced within the less obvious cases of coercive control highlighting that many members of the public may not recognise coercive control in its more subtle forms or seek support early on within an abusive relationship.

Research has suggested that first responders such as police officers tend to rely on objective measures of risk of harm rather than considering the wider context of the situation. “To date, it appears the focus in developing risk assessment tools has been on individual risk factors and their summation and/or weighting, as opposed to applying theories of abuse in intimate relationships to understand how combinations of factors may represent particular patterns of abusive behavior” (Myhill and Hohl, 2016, p.4491). This may explain why the general public who participated in the present study were much more likely to agree that behaviours should be reported to the police and would be viewed by the police as criminal when the behaviours were obvious as opposed to less obvious. Myhill & Hohl describe coercive control as the ‘golden thread’ running through risk identification and assessment. That said, as Muehlenhard and Kimes (1999) note, “What counts as "violence" is socially constructed” (p.234), the process of labelling particular behaviours as abusive is not straightforward, and to only identify coercive control when it is obvious and extreme is too late. The development and implementation of legislation criminalising non-physical abuse is a welcome development but to work in practice will require “nuanced understandings of when behaviour has become criminal” (Killean, 2020, p.13). Victim perceptions and instincts are often the best indicator of their own risk (Myhill and Hohl, 2016). Notably, given the size of the current survey sample, it can be assumed that at least some respondents may have also experienced the behaviours described.

### Implications & Directions for Future Research

A key strength of the present study was the manipulation of the obviousness of the coercive control behaviours portrayed in the scenarios presented. This manipulation was a necessary feature given that coercive control behaviours often begin subtly and escalate over time (Stark, 2013). It would seem intuitively important that the introduction of the coercive control as a criminal offence in any country should be accompanied by a public awareness campaign focusing on what coercive control is and what it means, and signposting victims and their friends and family to appropriate courses of action and sources of support. This may also enable victims to use appropriate descriptions and terminology to report coercive control. Such campaigns have been reported as a strategy to prevent domestic violence (Gadomski et al., 2001) though there is a paucity of evidence regarding their effectiveness (Campbell & Manganello, 2006) and to our knowledge they have not included education about coercive control. The current research has provided a baseline of public attitudes towards coercive control prior to any future awareness campaigns. A comparable survey post awareness campaign could be implemented to allow for future comparison.

Given the noted lack of awareness and understanding of coercive control among younger age groups, it is important to further explore this with those under 18 years of age. This should include adoption of scenarios which are relatable to younger age groups (e.g. inclusion of online abuse tactics). Additionally, early educational interventions which can be embedded into the curriculum which focus on the development of both healthy and unhealthy relationships should be considered in order to forearm those potentially most at risk of IPV. Such education programmes focusing of sex and relationships have a long history (Meyer & Stein, 2004; Pound et al., 2017) but again, to our knowledge, rarely include information about coercive control. The inclusion of information on coercive control and the continued evaluation of such programmes is important in terms of future research.

### Limitations

The level of analysis available from the current data limits in depth exploration of the societal reasons that may underlie the gender bias towards male and female victims of IPV among this population. That said, recent evidence following from a Fundamental Rights Survey (FRA, 2021) on crime, safety and victim rights supports this notion, reporting that people are generally less willing to intervene when a female physically assaults a male in comparisons to male violence against a female. Further in-depth qualitative exploration is needed to uncover if specific gender stereotypes underlie these attitudes. However, the findings do provide a unique insight in to the level and individual level correlates of knowledge and understanding of coercive control in society.

Given the nature of population surveys, there is always a degree of uncertainty as to the accuracy of the responses received. We cannot rule out the possibility of social-desirability bias in the answers given, which may have led to an overestimation of the level of awareness of coercive control. The limitations with the questions used to ascertain knowledge of coercive control should also be acknowledged. It is difficult to fully elicit participant’s awareness of coercive control in a survey. The question asked if they thought it was commonplace but does not clarify how common they thought it was. Future research should explore differences in perceptions of the prevalence of coercive control across different genders and other groups. Also asking if someone has heard of the term coercive control is a quite basic measure of awareness. Other questions could be asked to unpick this further, but to properly explore awareness, qualitative methods would allow for a deeper understanding of awareness.

Finally, attitudes to domestic abuse vary across cultures. Due to the limited diversity in the Northern Ireland population, it was not possible to analyse data to compare levels of knowledge and understanding across different ethnic groups. Future research should therefore extend to other countries to explore differences in those from different cultural backgrounds.

## Conclusions

The experience of domestic abuse is seldom the result of an isolated incident. Coercive control as a form of domestic abuse generally becomes apparent when associated behaviours develop into a pattern over time, by which stage the importance of identifying the early ‘minor’ acts of coercion and control have become clear (Stark, 2012). This type of abuse is often hidden in plain sight, with consequences devastating victims and lasting years after the abuse has ended. Current study findings indicate that while some respondents are aware of the term coercive control, a significant number do not know what this means and are therefore unlikely to recognise the signs of this type of abuse beyond obvious and blatant acts of harmful behaviour. Our results also show that male victims of coercive control are perceived as being at lower risk of harm, possibly due to gender biases in what behaviours are considered acceptable in relationships. While it is well understood that women are at greater risk of victimisation, this should not deter efforts to ensure there is appropriate awareness of risk amongst the wider public and access to support for all victims regardless of their personal demographics.

Research continues to reflect on the difficulty in operationalising coercive and controlling behaviour (Stark & Hester, 2019), but the effectiveness of early intervention as a form of prevention is well understood. Knowing the signs of a healthy relationship is an important mediator towards identifying unhealthy and harmful behaviours, as is knowing and navigating support services if they are needed. Likewise, it is imperative to have specialist training for police and advocacy services which enables them to recognise and respond to the defining features of coercive control, as well as ensuring a partnership approach which meets the needs of all victims. Current research and legislation developments across the UK and Europe have marked a critical moment for this field and an opportunity to set the groundwork for addressing domestic violence and abuse in all its forms. Awareness raising among populations should be the catalyst for change.
